# Comment from the Editor to the Special Issue: “Periodontitis: From Dysbiotic Microbial Immune Response to Systemic Inflammation”

**DOI:** 10.3390/jcm8101706

**Published:** 2019-10-16

**Authors:** Jan Oscarsson, Anders Johansson

**Affiliations:** Department of Odontology, Umeå University, S-901 87 Umeå, Sweden; jan.oscarsson@umu.se

**Keywords:** periodontitis, cardiovascular diseases, rheumatoid arthritis, *Porphyromonas gingivalis*, *Aggregatibacter actinomycetemcomitans*, inflammatory response

## Abstract

The human oral cavity contains a large number of different microbial habitats. When microbes from the oral indigenous flora colonize the interspace between the tooth and the connective tissue, they induce an inflammatory response. If the microbes are in sufficient numbers, and release components that cause an imbalance in the host inflammatory response, degenerative processes in the surrounding tissues are induced, ultimately resulting in periodontal disease. The disease progress depends on bacterial load, the composition of the microbial community, and host genetic factors. The two most studied periodontal pathogens, *Porphyromonas gingivalis* and *Aggregatibacter actinomycetemcomitans* express virulence factors, including proteases and exotoxins. Periodontal infections are also linked to the risk pattern of several systemic diseases. We would like to shed light on the mechanisms behind periodontitis and the associations of periodontal infections with systemic inflammation. Seven articles are included in this Special Issue and cover several pathogenic processes in the periodontal infection with capacity to cause imbalance in the host response. Highlights from each of the published papers are summarized and discussed below.

This Special Issue discusses the factors that induce a dysbiotic microbial periodontal immune response in periodontitis, which might result in a systemic inflammation. Könönen and co-workers [1] define periodontitis as an infection-driven inflammatory disease, by which the composition of the microbial biofilms play a significant role. Moreover, genetics and environmental or behavioral factors are involved in the development of the disease and its progression. The authors conclude that periodontal disease is multifactorial and the imbalance between tissue loss and gain can occur, due to various reasons, including aggressive infection, uncontrolled chronic inflammation, weakened healing, or all of the above simultaneously. Thus, successful disease management requires an understanding of the different elements of the disease at the individual level, and the design of personalized treatment modalities, including immunotherapies and modulators of inflammation.

In the second paper, Dahlén et al. [2] emphasize that the role of the classic, putative periodontal pathogens in the disease is still unclear, and the infectious nature of periodontitis today is in question. However, there is an enormous complexity and variability that takes place, both within the dental biofilm communities and in the inflammatory response, which makes it challenging to disclose the actual roles of specific microorganisms in periodontitis. Inflammation in the gingiva (gingivitis) is a normal host tissue response, induced by commensal microorganisms and their released products (metabolites, endotoxins). The infectious nature of the microbes, and the extent to which the specific virulence factors induce a dysbiotic host response leading to an impaired tissue repair, remain unclear. Most of the factors discussed in terms of virulence (proteases, LPS, invasive ability, fimbriae, capsule, and leukotoxin), among the microorganisms that are commonly associated with periodontitis, should rather be termed microbial survival factors. One exception is the leukotoxin produced by *Aggregatibacter actinomycetemcomitans*, which highly leukotoxic genotype (JP2) best fulfils the designation of a periodontal pathogen in the human oral microbiota. Presence of the JP2 genotype in periodontally healthy adolescents has previously been shown to be a strong risk marker for a future development of periodontal attachment loss.

*A. actinomycetemcomitans* is a facultative anaerobic Gram-negative bacterium that induces cellular and molecular mechanisms, and is associated with the pathogenesis of periodontitis. This bacterium is present in the oral cavity of a large proportion of the human population. However, its association to disease is mainly limited to young carriers. In their review, Oscarsson and co-workers [3] discuss virulence mechanisms that enable *A. actinomycetemcomitans* to evade the host response. These properties include invasiveness, secretion of exotoxins, serum resistance, and release of outer membrane vesicles. It is today hypothesized that the virulence characteristics of *A. actinomycetemcomitans* allow this organism to induce an immune subversion that tips the balance from homeostasis over to disease in oral and/or extra-oral sites. Hence, in order to prohibit the negative systemic consequences that are associated with periodontitis, successful treatment in an early phase of the disease is fundamental. The development of specific diagnostic tools for the assessment of periodontal pathogens and inflammatory components in the saliva of young individuals might make it possible to prevent the disease before its onset.

Antigens, released from the periodontal bacteria, activate both, a local and systemic immune response. These responses normally prevents microbial invasion deeper into the tissues surrounding the teeth, or into circulation. The work by Pietiäinen and collaborators [4] focuses on the immune response against bacteria occurring in apical periodontitis, an inflammatory disease that affects the tissues surrounding the apex of the tooth, which is initially triggered by oral pathogens infecting the root canals. The study investigated serum and saliva antibodies against several oral pathogens associated with apical periodontitis, and the role of cross-reactive antibodies in the disease. The authors concluded that this form of periodontitis associates with adaptive immune responses against both bacterial- and host-derived epitopes, in line with other forms of periodontitis. In addition, their results indicate that salivary immunoglobulins could be useful biomarkers in oral infections, including apical periodontitis, a putative risk factor for systemic diseases.

A number of host-derived risk marker candidates, associated with periodontal inflammation, have been the focus of many different experimental studies. The triggering receptor, that is expressed on myeloid cells-1 (TREM-1), a modifier of local and systemic inflammation, has been studied by Bostanci and co-workers [5]. Bacterial infections can upregulate the membrane-bound and soluble forms of TREM-1, which in turn amplifies inflammation. The blockade of TREM-1 engagement by either soluble forms of TREM-1 or synthetic peptides reduces the hyper-inflammatory responses and morbidity. The result obtained in the present study demonstrated the involvement of TREM-1 in alveolar bone resorption during the course of experimental periodontitis in mice. TREM-1 reduced the RANKL/OPG osteoclastogenic ratio, presumably via the inhibition of IL-17. The authors suggest that a previously unidentified TREM-1-driven axis for inflammatory bone loss could be targeted via small-molecule antagonists for therapeutic intervention in human periodontitis.

An association between cardiovascular diseases (CVD) and periodontitis has been established over the past several decades. Grant and Jönsson [6] focus their review on the association between the oral microbiota and the most well-established mechanistic pathway by which the oral microbiota may modify CVD, namely via the nitric oxide (NO) synthesis pathway. Next generation sequencing has been used over the past two decades to gain deeper insight into the microbes involved, their location, and the effect of their removal from the oral cavity. Overall, these studies have demonstrated that there are nitrate and nitrite-reducing bacteria found in the mouth, and that their removal causes systemic effects, i.e., through a temporary increase in blood pressure. The authors have highlighted the role of the oral microbiota in the conversion of nitrate to nitrite and its importance to systemic balance. A deeper understanding of the role of oral microbiota will allow future interventions to proceed, including personalized medicine approaches, and potentially reduce the use of antimicrobials.

Another systemic disease associated with periodontitis is rheumatoid arthritis (RA). This is an autoimmune disease of unknown etiology, characterized by immune-mediated damage of synovial joints and antibodies to citrullinated antigens. Gómez-Bañuelos and co-workers [7] discussed the clinical and mechanistic evidence concerning the role of the common periodontal pathogens *A. actinomycetemcomitans* and *Porphyromonas gingivalis* in RA pathogenesis. Both these pathobionts exhibit virulence mechanisms that promote citrullination of proteins, which indicate a possible involvement in the formation of the RA-associated autoantibodies against citrullinated antigens. For example, *P. gingivalis* produces a peptidylarginine deaminase that converts arginine to citrulline, and the *A. actinomycetemcomitans* leukotoxin activates neutrophil degranulation, which results in release of extracellular net-like structures that contains citrullinated proteins. The authors concluded that these oral pathobionts, together, give an opportunity to understand whether bacterial-associated citrullination is a mechanism involved in RA pathogenesis. These discoveries have the potential to be used in the implementation of future preventive interventions in RA. 

We can conclude that the articles in this Special Issue give a comprehensive overview of the complex interplay between the oral microbiota and the host response, which can induce the degenerative processes in the tooth supporting tissues, ultimately resulting in periodontitis. Increased knowledge about these biological processes will contribute to the development of improved preventive and treatment strategies for periodontal disease. New biomarker candidates, that are the potential targets for therapeutic strategies, are continuously discovered and could make personalized dentistry into a reality in the future.
